# High cost or frequent attender – both spend resources, but are they linked to work disability? A cohort study from occupational health primary care in Finland

**DOI:** 10.1186/s12913-020-05330-2

**Published:** 2020-05-24

**Authors:** Tiia T. M. Reho, Salla A. Atkins, Nina Talola, Markku P. T. Sumanen, Mervi Viljamaa, Jukka Uitti

**Affiliations:** 1 Tampere University, Faculty of Medicine and Health Technology, PB 100, FI-33014, Tampere, Finland; 2 Pihlajalinna Työterveys, Tampere, Finland; 3 Tampere University, New Social Research and Faculty of Social Sciences, Tampere, Finland; 4 Department of Global Public Health, Karolinska Institutet, Stockholm, Sweden; 5 Finnish Institute of Occupational Health, Tampere, Finland; 6 Clinic of Occupational Medicine, Tampere University Hospital, Tampere, Finland

**Keywords:** Primary health care, Patient acceptance of health care, Occupational health services, Cohort studies

## Abstract

**Background:**

High use of services is associated with ill health and a number of health problems, but more information is needed on whether high use of services presents a risk for future pensions or disability. We aimed to investigate if defining patients as high cost (HC) or frequent attenders (FA) was more useful in occupational health services (OHS) as a predictor of future disability pension (DP).

**Methods:**

This cohort study used medical record data from a large OHS provider and combined it with register data from the Finnish Centre for Pensions including disability pension decisions. A total of 31,960 patients were included and odds ratios for DP were calculated. Frequent attenders (FA10) were defined as the top decile of visitors according to attendance and high cost (HC10) as the top decile according to costs accrued from service use in 2015. Those patients that were not categorized as FA nor HC, but were eligible for the study were used as the control group (non-FAHC). The outcome measure (disability pensions) was analysed for years 2016–2017.

**Results:**

FA and HC did not significantly differ in their risk for disability pension. Both groups’ risk was higher than average users’ risk (adjusted OR 3.47 for FA10, OR 2.49 for HC10 and OR 0.33 for controls). Both HC10 and FA10 received half of their disability pensions based on musculoskeletal disorders, while for non-FAHC only 28% of pensions were granted based on these disorders. The groups overlapped by 68%.

**Conclusions:**

High utilizers (both FA10 and HC10) have an increased likelihood of receiving a future disability pension. The chosen definition is less important than identifying these patients and directing them towards necessary rehabilitation.

## Background

Use of healthcare services and subsequent healthcare spending are distributed unevenly in the population. Understanding of the phenomenon is crucial as top 10% of users can create 40% of the service demand [1, 2] and depending on the context, the top 5% accounts for 50% [3] and top 10% up to 70–80% of costs [4, 5]. In general, high utilizers have more physical and mental illnesses but also more social problems and overlapping multimorbidity [6, 7]. It appears that both illness related factors and personal and social characteristics affect service use [8]. Although settings, definitions and exact patient’s characteristics vary, studies demonstrate that high use of healthcare services relates to an increased need for care and coordination. High utilizers are a vulnerable group of patients with a number of difficulties. Thus, this group should be identified to plan their services better in order to improve their health, and to coordinate services to improve the continuum of care.

Identifying future and current high utilizers of healthcare requires analysis of existing healthcare use patterns. So far, various characterizations have been used to define these patients, including different cut offs for repeated use of services [9] and highest percentiles of cost [10]. International research indicates that patients defined as high-cost (HC), often have low socio-economic status, are older and have multiple chronic conditions and low perception of health [10, 11]; while frequent attenders (FA) frequently suffer from psychiatric problems, medically unexplained symptoms and injuries [1, 12]. These findings have mainly been from public or private primary care in settings where clientele varies in terms of ages and sociodemographic backgrounds [10, 13]. As working life sets demands to health and work ability but work has also beneficial health effects [14], studying the working population separately could yield new information about high cost and frequent attenders.

The organization of the Finnish occupational health primary care allows for studying the working population separately from the rest of the population. Primary care services in Finland are provided in three parallel service sectors: municipal, occupational health (OH) and private services. Preventive occupational health services (OHS) are legislated and primary care OH services are voluntary for organisations, though widely used, and covers up to 90% of employees [15]. The expense is directly covered by the employer and subsidized by an insurance paid by employers and employees. Previous work has shown that most employees use their OH unit as their sole primary care provider [16] in particular for issues affecting work ability [17]. The primary care provided should support the OH service preventive functions, most importantly prevention of working disability [18]. Identifying individuals at risk of future disability through medical record data collected on their primary care visits allows for directing preventive measures, such as early rehabilitation plans, to these patient groups. HC or FA definitions have not been used thus far in OHS to identify patients at high risk, but identifying high utilisers could have value for service design. However, whether HC or FA has better predictive value of future disability, is unclear. The ultimate aim of OHS is to prevent disability and disability pensions (DP), and thus we should examine which definition predicts future disability better.

The aim of this study is to examine two definitions of patients who consume more OHS resources than others: high cost and frequent attenders, and to examine how these two categories of patients differ in their characteristics and their risk of future disability pensions.

## Methods

### Study design

This is a cohort study combining routine electronic medical record data with register data.

### Setting

The study used routine medical record data from a large OHS provider, Pihlajalinna. It operates nationwide in Finland serving both municipal and private employees from a variety of industries, and the clientele is fairly representative of the Finnish working population. At the end of 2015 Pihlajalinna had 37 OHS units around the country in both urban and rural areas. In the OHS physicians, nurses, physiotherapists and psychologists mostly with OH specialization provide both legislated preventive functions and primary care for their clients. The preventive functions of the OHS are mandatory and can be complemented by primary care, which is available to approximately 90% of employees. Both preventive and primary care functions of the OHS are paid by the employer, and free for the employees [15].

Data collected also includes received decisions on disability pension benefits from the Finnish Centre for Pensions (FCP). A disability pension (DP) may be granted in Finland to individuals with diminished work ability caused by an illness. For a full DP work ability must be reduced by at least 3/5. This DP is permanent and leads to withdrawal from the work force.

### Data collection

All data on face-to-face OH primary care contacts from 2015 were collected. This data were used to determine the different study groups and the groups were determined according to service use in 2015. Visits to physicians, nurses, psychologists and physiotherapists and consultations with other medical specialists were extracted from electronic medical records by Pihlajalinna. The data included visit details such as diagnosis and professional visited, but also the cost (spending) of each consultation and sickness absence days given. In addition to this process data, the employee age, gender, and the employer’s main industry and company size were extracted. Pihlajalinna collected the data and sent the pseudonymised data to Tampere University. Data obtained from the FCP contained decisions on DPs (years 2016–2017) and the diagnostic codes associated with the decision [19]. The data were collected at FCP based on social security numbers provided by Pihlajalinna and the data on pensions and were sent pseudonymised to Tampere University.

At the end of 2015 Pihlajalinna had 68,370 employees on their primary care contract list. Approximately three quarters of these (45999) conducted a primary care visit in 2015. We included all patients that were aged 18–68, whose employers had bought a primary care plan, and who had had at least one curative face-to-face contact with an OHS primary care unit in 2015. When defining FA and HC, we excluded all general medical examinations, mandatory occupational safety examinations or those that were not conducted face-to-face (telephone calls, prescription renewals). There are no inpatient periods in the OHS. Only the first (i.e. the main) diagnosis recorded for the visit was considered in the analysis. The recording according to International Classification of Diseases 10th edition (ICD-10) is mandatory and was missing in only 1% of visits.

### Data analysis

The categorization into different groups (FA, HC, FAHC and non-FAHC) were done based on service use in 2015. FA were defined as the top decile of attenders as in previous studies [2, 9], which meant 8 or more visits a year [20]. We used visits to physicians, nurses, physiotherapists and psychologists to define frequent attenders [20]. The top decile was defined as the 10 % of those patients that used services most, in 2015 (belonged to the highest 10% of patients visiting the OHS). We defined HC as those patients that ranked in the top 10% according to their annual spending in the OHS [10, 11]. All primary care costs (excluding laboratory and imaging costs) created in the OHS in 2015 were summed to define the HC-group [21]. Laboratory and imaging costs were excluded as they could be initiated by health check-ups and not by patient need. The same services were included in defining FA and HC. We also used to the combination definition (FAHC) for those patients that were in the top decile in both categories. The FAHC represent those patients that were both high cost and frequent attenders and includes same patients as the previous groups. As a control group we used patients that were neither FA10 nor HC10 in the study year (non-FAHC). These were patients that had used services in 2015 (had at least one face-to-face primary care visit to the OHS in 2015) but could not be considered FA nor HC. They belonged to the 90% of patients using services less than the highest decile (measured by both frequency of visits and costs).

The study population was divided by sex and age for characterization. Employer industries were categorized according to Statistics Finland/ Statistical classification of economic activities in the European Community (TOL2008/Nace Rev2). We characterized the data using frequencies and chi square –tests. When examining multimorbidity we used the Mann-Whitney U -test. The data were carefully revised and any outliers were checked. No extreme outliers were found in the data. The main outcome measure in the analysis was permanent DP as registered on the statistics obtained from the FCP. We included pension decisions given in 2016–2017. Odds ratios (OR) with 95% confidence intervals (CI) were calculated for the outcome measure for different FA groups. The results were adjusted for patient age, sex and employer industry and analysed separately. We also used logistic regression to analyze which characteristics were associated with the high use status groups (FA10, HC10, FAHC). Sickness absences for different groups were analysed using logistic regression. Hosmer and Lemeshow goodness-of-fit test was used to evaluate the fitness of the model. Statistical analyses were conducted at Tampere University using IBM SPSS Statistics and R-program. In all analyses *P* values less than 0.05 were considered statistically significant.

### Ethical considerations

Pirkanmaa hospital district ethics committee (ETL R16041) and the National Institute of Health and Welfare (THL/556/5.05.OO/2016) approved the study. According to Finnish legislation, individual consent was not needed since this a register based study, where no individual participant could be identified.

## Results

**Fig. 1:**
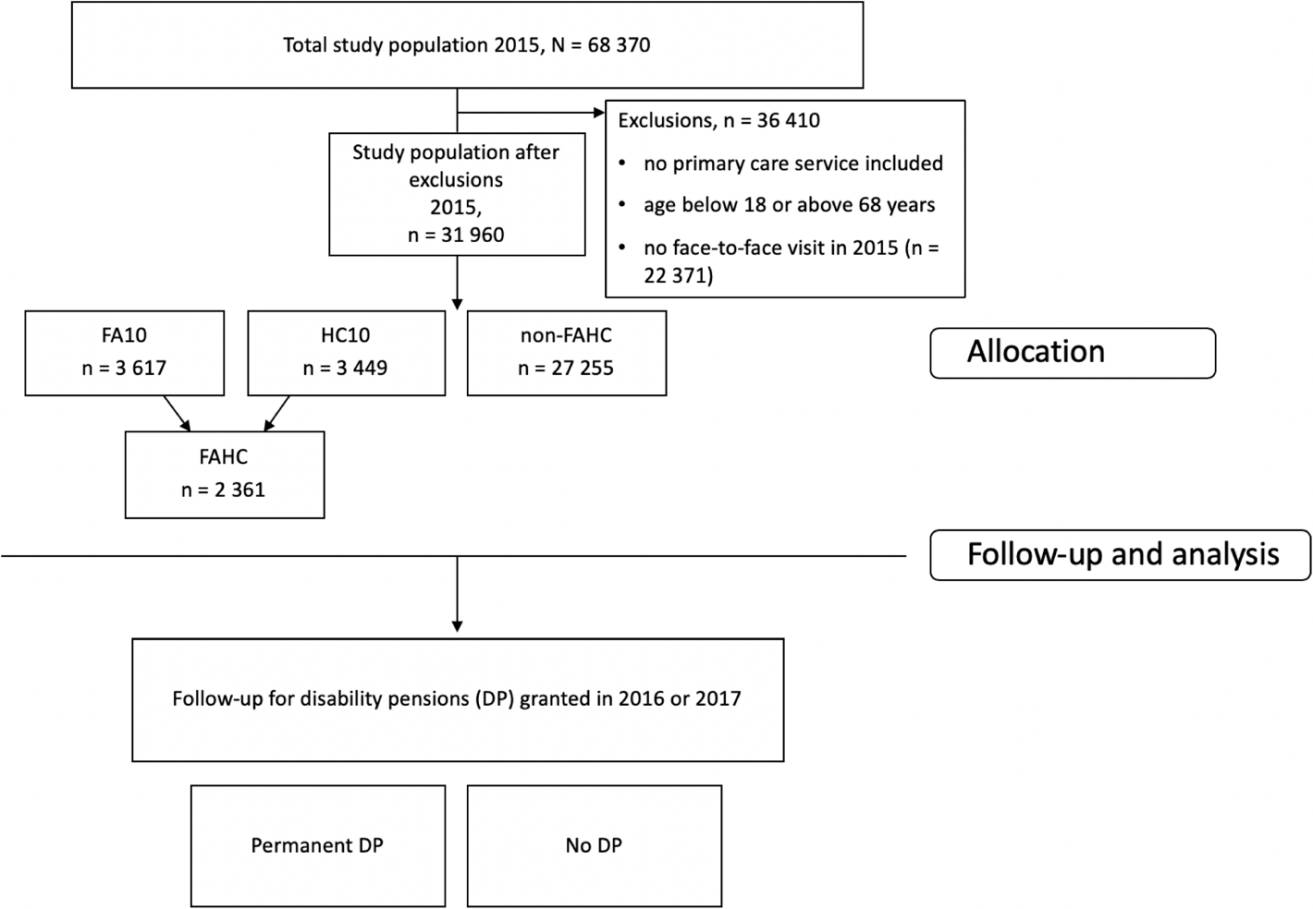
Flow diagram of the study population. HC10 = High cost 10%, being in the highest decile in 2015. FA10 = Frequent attender 10%, being in the highest decile in 2015. FAHC = Combination group of patients that belong to both FA10 and HC10 -groups. Non-FAHC = The control groups, the patients that were neither HC10 nor FA10

In 2015, Pihlajalinna Työterveys had 68,370 clients. Of these, 45,999 had contacted Pihlajalinna during 2015. The study population constituted 31,960 patients who met the inclusion criteria (Fig. 1). In 2015 patients visited Pihlajalinna Työterveys on average 3.7 times and the average cost per person was 213 €. Of the total visitors, 3617 were categorized as FA10 and 3449 as HC10. The average cost for the HC10 group was 706 € while for FA10 it was 602 €. The combination group of FAHC constituted 2361 patients (that were both FA10 and HC10) and their average cost was 749 €. The average cost for the non-FAHC group was 142 €. The 2361 patients in the FAHC group represent patients that were both high cost and frequent attenders. These groups overlap with 68% of patients included in both groups. Table 1 below presents the demographics of the study groups. There are more females in all high use groups compared to other users, and the proportion is greater with HC10 and FAHC groups than FA10. The age groups above 45 are accentuated in the HC10s.

**Table 1:** Characteristics of frequent attender 10% (FA10), high cost 10% (HC10) and patients in both categories (FAHC) compared with non-frequent attender and non-high cost users (non-FAHC), *N* = 31,960

	FA10 *n* = 3617		HC10 *n* = 3449		FAHC *n* = 2361		non-FAHC *n* = 27,255	
Characteristics	n	%	n	%	n	%	n	%
Sex								
Male	1811	50	1622	47	1120	47	15,994	59
Female	1806	50	1827	53	1241	53	11,261	41
Age								
18–34	840	23	654	19	505	22	8158	30
35–44	908	25	828	24	589	25	6502	24
45–54	983	27	1032	30	666	28	7288	27
55–68	886	25	935	27	601	26	5307	19
Company size								
0–10	227	6	304	9	139	6	3851	14
11–50	862	24	861	24	547	23	7735	28
51–250	1111	31	1054	31	756	32	6752	25
> 250	1417	39	1230	36	919	39	8917	33
Professionals visited in 2015 *								
Doctor	3609	100	3436	100	2358	100	24,790	91
Nurse	2068	57	1512	44	1290	55	7804	29
Physiotherapist consultation	1489	41	1073	31	931	39	2726	10
Psychologist consultation	232	6	274	8	186	8	737	3
Specialist consultation	901	25	998	29	713	30	1939	7
OH collaborative negotiation								
No	3294	91	3150	91	2105	89	27,032	99
Yes	323	9	299	9	256	11	223	1
Permanent disability pension (2016–2017)	49	1	52	2	43	2	96	0
Industry								
Manufacturing	1398	39	1070	31	864	37	8304	31
Wholesale and retail trade; repair of motor vehicles and motorcycles	313	9	358	10	198	8	3054	11
Professional, scientific and technical activities	183	5	193	6	123	5	1610	6
Public administration and defense; compulsory social security	346	10	305	9	231	10	2043	7
Human health and social work activities	433	12	417	12	294	12	2461	9
Others	944	26	1106	32	651	28	9783	36

The results of the study are presented according to the latest industry classification system from 2008 that is based on the Statistical classification of economic activities (NACE Rev. 2)

*A patient might have visited more than one professional

As expected, all diagnostic groups are associated with high use of services. However, this association is enhanced in diseases of the musculoskeletal system (M00–M99) and mental disorders (F00–F99) (Table 2). The differences between the high use groups are significant in mental disorders and injuries. The association with mental disorders was stronger for FA10 than HC10. On the other hand, diseases of the musculoskeletal system are more closely associated with FAHC than HC10. When suffering from diseases of the musculoskeletal system or mental disorders one was likely to be both frequent attender and high cost. All high use status groups (FA, HC, FAHC) had a median of three different ICD-10 diagnoses while the group of non-FA had a median of one (*p* < 0.001).

**Table 2:** Diagnoses associated with frequent attender 10% (FA10), high cost users (HC10) and both FA10 and HC10 (registered for physician consultations, adjusted for age, sex and industry), *n* = 29,380

	FA10		HC10		FAHC (FA10 & HC10)	
ICD-10	OR	95% CI	OR	95% CI	OR	95% CI
A00-B99 Certain infectious and parasitic diseases	2.43	2.18–2.71	2.34	2.09–2.63	2.67	2.36–3.03
F00-F99 Mental and behavioural disorders	4.34	3.96–4.76	3.52	3.19–3.87	4.16	3.74–4.62
G00-G99 Diseases of the nervous system	2.74	2.44–3.08	3.41	3.04–3.83	3.32	2.91–3.77
I00-I99 Diseases of the circulatory system	1.82	1.63–2.03	2.09	1.88–2.33	2.14	1.89–2.43
J00-J99 Diseases of the respiratory system	2.47	2.30–2.66	2.31	2.15–2.49	2.98	2.73–3.26
K00-K93 Diseases of the digestive system	2.45	2.18–2.75	2.76	2.45–3.10	2.82	2.47–3.21
L00-L99 Diseases of the skin and subcutaneous tissue	2.18	1.97–2.41	2.22	2.00–2.46	2.45	2.19–2.75
M00-M99 Diseases of the musculoskeletal system and connective tissue	4.09	3.79–4.41	3.90	3.61–4.21	4.67	4.24–5.13
R00-R99 Symptoms, signs and abnormal clinical and laboratory findings, not elsewhere classified	2.92	2.69–3.17	3.02	2.78–3.28	3.41	3.11–3.75
S00-T98 Injury, poisoning and certain other consequences of external causes	3.11	2.87–3.38	2.03	1.86–2.22	2.42	2.20–2.68

*OR* Odds ratio, *CI* Confidence interval

The diagnostic groups were used as dummy variables (No = reference group = 1.00)

ICD-10 = International Classification of Diseases

The 10 largest ICD-10 groups are presented

Other factors associated with FA10 and HC10 are shown in Table 3. Being female was associated with both FA10 and HC10. There is an association of specialist consultation and HC10 which appears enhanced compared to FA10. All high use status groups have more permanent disability pensions than the control group (Table 1).

**Table 3:** Factors associated with frequent attender and high cost status, *n* = 31,960

	Frequent attender 10% (FA10)				High cost user 10% (HC10)				Frequent attender High cost (FAHC)			
	Crude ratios		Adjusted ratios*		Crude ratios		Adjusted ratios*		Crude ratios		Adjusted ratios*	
Factor	OR	95% CI	OR	95% CI	OR	95% CI	OR	95% CI	OR	95% CI	OR	95% CI
Sex												
Male	1.00		1.00		1.00		1.00		1.00		1.00	
Female	1.38	1.29–1.49	1.41	1.31–1.51	1.59	1.48–1.71	1.59	1.48–1.71	1.53	1.41–1.67	1.55	1.42–1.69
Age												
18–34	1.00		1.00		1.00		1.00		1.00		1.00	
35–44	1.33	1.21–1.47	1.34	1.21–1.48	1.58	1.42–1.76	1.60	1.44–1.78	1.43	1.26–1.61	1.44	1.27–1.63
45–54	1.27	1.15–1.40	1.26	1.14–1.39	1.76	1.59–1.95	1.77	1.60–1.96	1.43	1.27–1.61	1.43	1.26–1.61
55–68	1.55	1.41–1.72	1.54	1.41–1.72	2.17	1.95–2.41	2.16	1.95–2.40	1.74	1.54–1.96	1.72	1.52–1.95
Specialist consultation	3.90	3.57–4.25	3.89	3.56–4.24	5.05	4.64–5.50	5.13	4.70–5.59	4.88	4.43–5.38	4.87	4.41–5.37

* Adjusted for age, sex and industry when possible, OR = Odds ratio, CI = Confidence interval, 1.0 = reference group

All high use status groups (FA10, HC10 and FAHC) had more long sickness absences than non-FAHC. The results show 53% of FA10, 37% of HC10 and 56% of FAHC groups had a sickness absence longer than 15 days. Table 4 shows characteristics associated with sickness absences over 15 days in different groups. Female sex and morbidity (measured by the number of different diagnoses given by a physician) were associated with FA status in sickness absences over 15 days.

**Table 4:** Sickness absences > 15 days associated with FA10, HC10 and FAHC status in logistic regression model (adjusted for age, field of industry and cancer dg (C00-C97) and number of different ICD-10 diagnoses given by physicians), *n* = 16,111

	FA10 vs. Non-FA10		HC10 vs. Non-HC10		FAHC vs. Non-FAHC	
	OR	95% CI	OR	95% CI	OR	95% CI
Sex						
Male	**1.00**		**1.00**		**1.00**	
Female	**1.32**	1.01–1.72	**1.59**	1.23–2.05	**1.57**	1.18–2.09
Number of different ICD-10 diagnoses given by physicians	**1.91**	1.77–2.07	**1.67**	1.55–1.79	**1.87**	1.73–2.04

Being FA10, HC10 or FAHC increases the likelihood of receiving DP in the following 2 years as seen in Table 5. In the adjusted ratios the differences between the groups are marginal but the association with FAHC status appears stronger than the other high use groups. The increased risk turned into cases would mean an additional 35, 36 and 40 disability cases for the status FAHC, FA10 and HC10 respectfully.

**Table 5:** Factors associated with permanent disability pension (DP) for frequent attender 10% (FA10), high cost 10% (HC10) and both FA10 and HC10 (FAHC), *n* = 31,960

	Permanent disability pension			
	Crude ratios		Adjusted ratios*	
Factor	OR	95% CI	OR	95% CI
Sex, Female	0.98	0.71–1.35		
Age	1.17	1.14–1.20		
FA10**	3.69	2.63–5.19	3.47	2.46–4.91
HC10**	4.26	3.05–5.97	3.50	2.49–4.93
FAHC**	4.93	3.46–7.03	4.55	3.17–6.54
non-FAHC	0.28	0.20–0.39	0.33	0.24–0.46

*OR* Odds ratio, *CI* Confidence interval

*Adjusted for sex, age and industry

** Hosmer and Lemenshow < 0.05

When looking at the diagnoses leading to permanent DP majority of high cost and frequent attenders’ disability pensions are due to diseases of the musculoskeletal system (49, 50 and 51% for FA10, HC10 and FAHC respectively) while for non-FAHC the proportion was 28%. The distribution of diagnoses leading to permanent DP is more widespread for non-FAHC and they appear to have particularly more diseases of the circulatory system leading to DP (17%) than high cost or frequent attenders’ groups (less than 6%).

The cost of HC10 group compared to any other user was due to physiotherapist visits (9.7% for HC10 and 1.5% for others) and other medical specialist visits (4.0% for HC10 and 1.9% for others). On the other hand, the cost due to physician visits was smaller for HC10 (76.1%) compared to others (82.0%). As a whole, when laboratory and imaging costs were excluded, most costs were due to physician visits.

## Discussion

This study found that high use of services, whether measured by attendance rates or costs, is associated with disability pensions in the following 2 years. The associated risk is fairly similar regardless of the chosen definition. In addition, the characteristics between the high cost and frequent attenders’ groups are fairly similar. These findings suggest that the chosen definition is less important than taking action in identifying these groups and planning necessary measures to maintain work ability.

To our knowledge this is the first study to analyze differences in disability pension risks between high cost and frequent attender groups. We chose to analyze the differences in relation to disability pension as this reflects the risks linked to high use of services; it is not only a question of resources and their management, but high use of services reflects health related problems that demand attention. The elevated risk of future disability pension of both these groups indicates that the service demand is real and should be taken seriously. On the other hand, understanding which definition reflects DP in the near future better is essential for deciding which definition to use in identifying high utilizers in OH services. This study provides data for service providers to choose the definition that indicates risk of disability more accurately than the other. Based on this OH services can identify candidates for rehabilitative actions. Prevention of future disability is one of the key aims of OHS [18] and service use is a possible marker in identification of those individuals that need more supportive measures. Our results indicate that the differences between the definitions in this context are minimal between frequent attender and high cost users – the combined definition (FAHC) appears to have the highest likelihood of having a future disability pension, but the differences between the groups are relatively small. Based on our study, it appears less important whether high use is defined according to attendance rate or costs, and the choice can be made based on data more easily accessible.

When looking at the diagnoses leading to permanent disability pension the high utilization groups differ from each other only slightly. However, they differ substantially from the control group (non-FAHC) portraying more musculoskeletal disorders leading to disability pension and fewer of any other diagnostic group leading to disability. There is little previous research on the associations of high use of services and disability pensions. Existing studies suggest that frequent attendance is associated with being on disability pension [22, 23]. In addition, a Swedish study from general practice setting showed [24] that frequent attenders are more likely to be on long sick leave or on disability pension but the diagnoses leading to this withdrawal from the work force were unknown. Our study indicates that although the differences between the different high utilizer groups are small, they differ from the other users of OH services by having more musculoskeletal disorders severe enough to cause permanent disability. This indicates that in particular high utilizers suffering from musculoskeletal disorders should be identified and that careful evaluation of necessary rehabilitative actions is needed.

When examining the visits, diseases of the musculoskeletal system are associated with frequent attenders and the combination group, FAHC, in particular. The connection with mental disorders is stronger also for frequent attenders than for high cost users. To our knowledge there are no previous studies comparing differences in diagnoses of frequent attenders and high cost users. Previous studies have shown that frequent attenders are prone to injuries [12] and our study indicates that the association to injuries is stronger for frequent attenders than high cost. On the other hand, the association of high cost and neurological symptoms has been described earlier [21, 25]. Our results add to this by showing that the association is stronger for high cost than frequent attenders. As expected, multimorbidity was present in both FA and HC groups.

As a whole the HC10 group constituted 37% on all the costs while the FA10 group constituted 33% of the costs. These groups overlapped so that 68% of frequent attenders were also high cost (FAHC, *n* = 2361). The average costs for FA10 or HC10 was four to five times higher than the average users’ cost. Comparison between the high utilizer status groups indicates that when laboratory and imaging costs are excluded, the differences between the groups in costs and background characteristics are rather small. There are more females in high cost and combination group and female gender is associated with being high utilizer in general. A previous study inspected the association of FA-status with female gender in OH services [20] and this study verifies the finding with also high cost and combination group in OHS. However, previously there are differing results regarding sex and high use of services depending on the setting [6, 21, 25]. Being older is associated with high cost in particular [21, 25] and our study verifies the association of older age with high cost also in OHS. Specialist consultations are more expensive than usual visits in the OH services, which can explain the association of HC10 with specialist consultation, a connection also shown previously [25]. Specialist visits may also generate follow-up visits and further examinations, which may add to this connection.

The strengths of our study are the large study population representing a variety of industries and urban and rural areas around Finland. Thus, the study population is fairly representative of the working population in Finland. Although we used data from one service provider, it allows for comparing different definitions. In addition, the amount of missing data was minimal. On the other hand, we were not able to include laboratory and imagining costs as we could not trace if they were associated with mandatory safety check-ups part of preventive care, which means that the groups might include different patients if these costs were included. As disability pensions is a fairly uncommon endpoint for any of the groups, this should be kept in mind in interpretation of the results. The goodness of fit of the model used in the analysis is not the best possible, but this analysis was chosen because of the familiarity and familiar interpretation. It is well established that sickness absences are closely associated with risk of future disability. In the analyses, this was not taken into account for any of the groups. We have previously detected that the disability pension risk associated with frequent attendance is closely linked to long sickness absences, which are more common for frequent attenders [26]. Next step should be to study whether already existing intervention strategies [27] are of use for high service utilizers or should there be targeted measures for these groups.

## Conclusions

Service use indicates risk of disability pension in the future and allows identification of groups that might benefit from rehabilitation. Defining these factors that are associated with future disability and might indicate rehabilitative needs prior to sickness absences, allows identification of these groups and constructing early rehabilitative and treatment plans. It is not nearly enough that we identify high utilisers from other patients, but we need to use this to enhance their services and coordination. At least in the OHS the aim should be to identify the individuals that might benefit most from rehabilitation and care coordination.

Service use could also be used as a marker for rehabilitative needs in addition to sickness absences allowing for early interventions. It appears less important whether high use is defined according to attendance rate or costs.

## Data Availability

The data that support the findings of this study are available from Pihlajalinna Työterveys but restrictions apply to the availability of these data, which were used under license for the current study, and so are not publicly available. Data are however available from the authors upon reasonable request and with permission of Pihlajalinna Työterveys.

## Abbreviations

CI: Confidence interval
FA10: Frequent attender 10% (patients in the top decile of annual visits to healthcare professionals)
GP: General practice
MUPS: Medically unexplained physical symptoms
OH: Occupational health
OHS: Occupational health services
OR: Odds ratios
HC: High cost (patients that ranked in the top 10% according to their annual spending in the OHS)
DP: Disability pension
FCP: Finnish Centre for Pensions
ICD-10: International Classification of Diseases, 10th edition
